# Effectiveness of Intraoperative Neuromonitoring in a Patient Undergoing a One-Level Transforaminal Lumbar Interbody Fusion: A Case Report

**DOI:** 10.7759/cureus.35580

**Published:** 2023-02-28

**Authors:** Mark Michael, Michael Stark, Barrett Woods

**Affiliations:** 1 Orthopedic Surgery, Jefferson Health New Jersey, Stratford, USA; 2 College of Medicine, Lake Erie College of Osteopathic Medicine, Bradenton, USA; 3 Orthopedic Surgery, Jefferson Health New Jersey, Washington Township, USA; 4 Orthopedic Surgery, Rothman Orthopaedic Institute, Philadelphia, USA

**Keywords:** tlif, transforaminal lumbar interbody fusion (tlif), transforaminal lumbar interbody fusion, pedicle breach, drop foot, lumbar spine, neuromonitoring

## Abstract

Posterior lumbar interbody fusion (PLIF) and transforaminal lumbar interbody fusion (TLIF) are common modes of operative treatment of lumbar radiculopathy and spondylolisthesis. An integral part of these procedures is the appropriate placement of pedicle screws to ensure proper fusion. Breach of the medial cortex during pedicle screw fixation can potentially cause permanent impairment for a patient; significant technology and resources have been universally devoted to preventing this complication. Intraoperative neuromonitoring (IONM) is a frequently used tool by spine surgeons, which, along with fluoroscopy, is traditionally thought to reduce the incidence of neurologic injury. Unfortunately, IONM is not infallible and, in certain studies, has not been shown to decrease the risk of neurologic compromise. This case presentation details the clinical course of a 55-year-old who underwent an L4-5 TLIF. Despite benign electromyography recordings intraoperatively, the patient presented postoperatively with a new-onset left foot drop and a CT scan that confirmed bilateral L4 screw malposition with a breach of the medial cortex. We hope to further advance the discussion regarding the dangerous inconsistency of IONM in hopes of identifying a multimodal approach to avoid dreaded complications like this one in the future.

## Introduction

Posterior lumbar interbody fusion (PLIF) and transforaminal lumbar interbody fusion (TLIF) are common modes of operative treatment of lumbar radiculopathy and spondylolisthesis. The fusion is performed utilizing a single interbody cage as well as pedicle screws, which are placed either free hand, under fluoroscopic guidance, or by robot-assisted navigation systems [1].

Neurologic injury secondary to malpositioned pedicle screw placement has been studied both in vitro and in vivo [2,3]. Literature reveals that placement of malpositioned pedicle screws, with or without associated neurologic injury, occurs anywhere from 20% to 40%, and is often independent of the technique used [2,3]. For example, Kotil et al. performed a study on pedicle screw placement without fluoroscopic guidance and found their screw malposition rates were equivocal to rates reported in the literature even when fluoroscopic guidance was used [3]. Raynor et al. performed a retrospective review on 418 patients who had lumbosacral spinal fusions with pedicle screws using a freehand technique. All 2,450 screws in this study were placed under electromyography (EMG) monitoring followed by a postoperative CT scan to evaluate the accuracy of pedicle screw placement [4]. A total of 4.7% of screws stimulated a positive EMG response intraoperatively. Of those 4.7% with a positive EMG response at any threshold (2.5-10.0 mA), only 13.9% of the screws were true positives, meaning they had breached the medial cortex. There were also a few false negative findings. For example, 0.3% of the screws did not elicit an EMG response intraoperatively, yet were found to have breached the medial cortex on postoperative CT scans.

It is important to keep in mind that not every pedicle wall breach will result in a neurologic deficit. Nonetheless, due to the close proximity of the pedicles to the spinal canal and neural elements, neurologic injury is indeed a very realistic complication following lumbar fusion surgery [5]. Iatrogenic nerve injury from malpositioned pedicle screws is most often from penetration of the medial pedicle cortex. Castro et al. evaluated L1-S1 pedicle screw placement in 42 cadaver specimens and 30 patients and found that 29% of screws resulted in medial pedicle wall breach, with those of more than 6 mm of deviation leading to a postoperative neurologic deficit [2]. Additionally, Ghobrial et al. performed a systematic review evaluating iatrogenic neurologic injury following lumbar spine surgery. Looking at 12 studies from 2004 to 2015, the rate of neurologic compromise ranged from 0.46% to 17%, with the average being 5.7%. In 11 of the 12 studies, screw malposition was the cause of neurologic injury, despite the use of intraoperative neuromonitoring (IONM) [6].

IONM is a tool used in spine surgery to help reduce the incidence of neurologic injury. IONM includes somatosensory-evoked potentials (SSEPs), motor-evoked potentials (MEPs), and triggered EMG. Intraoperative triggered EMG was first introduced in 1992 by Calancie et al. [7]. He studied this technique in a porcine model in hopes to obtain an objective method to evaluate pedicle screw placement [7]. The goal of triggered EMG is to notify the surgeon if a screw has been misplaced through the pedicle cortex and is in contact with nerve tissue. Technically speaking, triggered EMG works by applying an electrical stimulus through the pedicle screw, which evokes an EMG response in a specific myotome corresponding to that spinal level being instrumented. The triggered EMG response can be compared to a threshold of values to help determine whether the screw is in a good position, and most importantly, whether a breach of the medial cortex has occurred. When the medial pedicle cortical wall is intact, current will not flow as easily through neural tissue. In comparison, when the medial wall is breached, the current can flow much more freely to neural tissue, therefore, producing a compound muscle action potential (CMAP) that triggers a neuromuscular response from the corresponding myotome [8].

Despite IONM being a common practice amongst spine surgeons, some studies have shown that EMG monitoring is not an appropriate screening tool to detect cortical breaches. A retrospective review by Ajiboye et al. identified 9,957 patients who underwent a posterior lumbar fusion with and without EMG. Comparing fusion surgery with and without EMG, a neurologic injury occurred in 1.36% of patients with EMG, and 1.34% of patients without EMG. Overall, they found that the risk of postoperative neurologic compromise after posterior spinal fusion was low and that using EMG may not affect the outcome [9]. However, EMG monitoring can be an appropriate tool to use in conjunction with intraoperative fluoroscopy and postoperative CT scans [4,10].

We report a case of a 55-year-old, obese male who underwent a minimally invasive L4-5 TLIF for spondylolisthesis. Despite having a normal intraoperative signal on neuromonitoring, he developed a postoperative left foot drop. On a postoperative CT scan, the left L4 screw demonstrated a medial breach of the pedicle wall. The goal of this case report is to bring forth more discussion on neuromonitoring and its efficacy in detecting true motor deficits. We also hope to investigate whether there have been other cases in which postoperative neurologic deficits occurred, despite normal IONM, so that perhaps we can understand why or when this is more likely to occur.

## Case presentation

**Figure 1:**
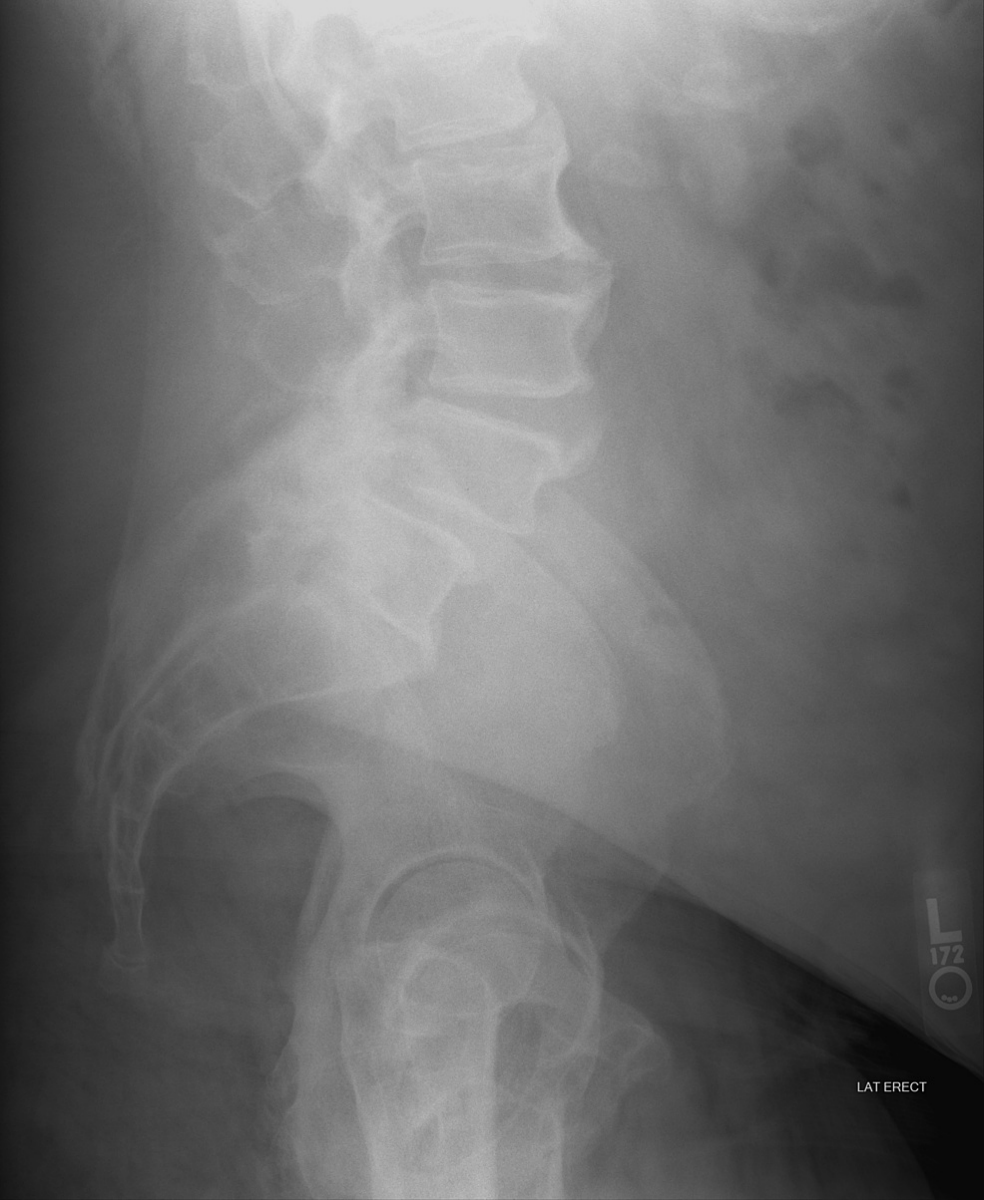
Preoperative lateral lumbar spine X-ray demonstrated an unstable L4-5 spondylolisthesis with significant facet hypertrophy at L4-L5.

This is a case report of a 55-year-old, obese male who presented with chronic lumbar back pain for approximately 10 years. Over the past several weeks, the patient noticed a progression and worsening of right-sided lower back pain and associated L5 radiculopathy and claudication symptoms. Physical examination revealed 4/5 strength of the right hamstring and extensor hallucis longus (EHL) with diminished sensation in the L5 distribution. Preoperative imaging demonstrated an unstable L4-5 spondylolisthesis, moderate central stenosis at L3-L4, significant facet hypertrophy at L4-L5, and severe foraminal and lateral recess stenosis, left greater than right (Figure 1). After months of failed conservative management (oral nonsteroidal anti-inflammatory drugs, physical therapy, and corticosteroid injection), the patient opted to undergo surgery.

Operative treatment consisted of a minimally invasive L4-5 TLIF. The patient received general anesthesia and was positioned prone on a Jackson table. IONM was provided throughout the procedure. Preoperative baseline neuromonitoring testing was performed prior to the incision. Baseline SSEPs showed normal signal latencies and amplitudes. Transcranial MEPs were also tested for all lower extremity muscle groups and found to be within normal limits.

A minimally invasive approach was performed with an incision centered over the L4-5 spinous processes. Screws were placed in the L4-5 pedicles under the guidance of fluoroscopy and IONM. Given the patient's large body habitus, fluoroscopy was obscured; thus, screw positioning relied more heavily on neuromonitoring.

A routine protocol for triggered EMG testing was followed. First, neuromuscular blockade was reversed with at least three of four twitches being present on stimulation. Next, the cathode, being a sterile alligator clip, was placed onto the rod-holding segment of the screw being tested. The anode, or subdermal needle, was placed in adjacent muscle tissue. Response images were captured with filter settings of 10 Hz to 20 kHz, amplifier sensitivity of 50 μV per division, and a 50-ms time-base. Constant current stimulation (rate: 1.0 Hz; duration: 0.3 ms) was delivered in an ascending method until a CMAP was obtained. Individual threshold levels were obtained for each screw. CMAPs were recorded using paired subdermal needle electrodes placed bilaterally in myotomes of the lower extremities, adductor longus for L2, vastus medialis for L3-4, tibialis anterior for L5, and medial gastrocnemius for S1. Previously established threshold data using a range rather than absolute values were used to determine accurate screw placement. The values are as follows: >8.0 mA = screw entirely in the pedicle; 4-8 mA = potential for pedicle wall defect; and <4 mA = high likelihood for pedicle wall defect and possible contact with a nerve or dural tissue. In our study, triggered EMG was recorded as 14 mA for the L4 left pedicle screw. The remaining three pedicle screws all recorded a stimulus greater than 8 mA as well. Additionally, there was no sustained loss of motor function with MEPs monitoring. To note, there was a transient increase in latency and decreased amplitude of the left L4 screw, which then spontaneously recovered. The remainder of the case was completed with no complications.

**Figure 2:**
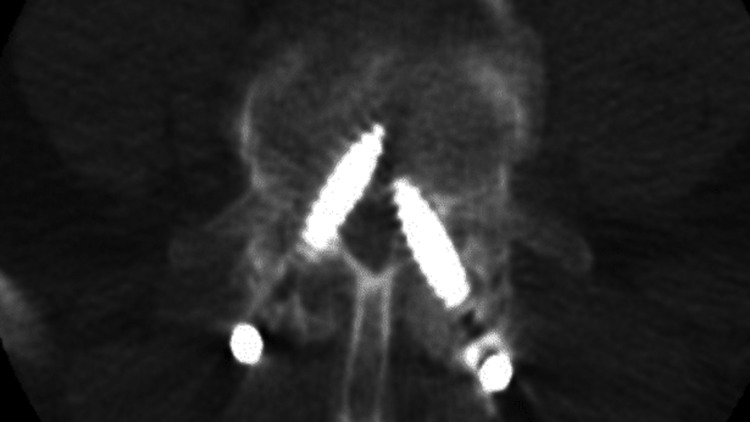
Postoperative CT scan demonstrating malposition of both L4 screws with a medial breach.

At the first postoperative physical exam, he had complete resolution of his preoperative right leg pain and his right-sided radicular symptoms had resolved. However, he had some slight left dorsiflexion weakness postoperatively, which progressed over the following one to two weeks, with eventual foot drop. Physical examination was notable for 1/5 strength of the tibialis anterior and EHL with diminished sensation in the left L4 distribution. Due to this new-onset neurologic deficit, a postoperative CT scan was obtained to assess the accuracy of pedicle screw placement. Malposition of both L4 screws with medial breach was seen on the CT scan (Figure 2). The patient was subsequently taken back to the operating room for revision surgery.

A midline incision was made with dissection down to L4-L5. A revision laminectomy was performed, and the entire L4 pars was removed. The medial breach of the L4 screws was then identified and removed under direct visualization. The L4 nerves were subsequently skeletonized bilaterally and inspected, and both were in continuity. A new screw trajectory was placed in the right L4 pedicle; however, the left L4 pedicle was blown with no identifiable significant purchase. Therefore, the decision was made to extend the fusion to L3. The remainder of the procedure continued without complication, including all screws being stimulated with EMG.

At his first postoperative visit following revision surgery, the foot drop persisted on the left, but the patient subjectively reported that sensation in the foot was returning. Physical exam was notable for 2/5 left dorsiflexion and diminished sensation in the L4 distribution on the left. At his subsequent follow-ups, his foot drop improved substantially over the next few months, increasing to 3/5 strength in dorsiflexion of anterior tibialis and EHL, with some mild associated diminished sensation on the lateral border of his foot. Unfortunately, after nine months, no further improvement was noted; therefore, the left foot drop was deemed chronic. The patient ambulates with a cane with the assistance of an ankle-foot orthosis (AFO) brace and was provided with disability at work given his body habitus and neurological deficits.

## Discussion

The purpose of this case report is to discuss neuromonitoring and its efficacy in detecting true motor deficits associated with posterior lumbar fusion. Stimulated EMG is an IONM technique used to help determine iatrogenic nerve injury resulting from malpositioned screws. Thus, the intraoperative EMG used in this case should have signaled the surgeon of a medial breach involving the L4 screw. Instead, the EMG for the left L4 screw had a stimulus of 14 mA. In our practice, any stimulus less than 8 mA raises concerns for possible malposition. Furthermore, a stimulus of 4-5 mA has a specificity of close to 100% for a pedicle wall defect [10]. In addition to the EMG stimulus, there was no sustained loss in motor function, which would have been expected with the patient’s postoperative clinical presentation of left foot drop. This raises the question as to how accurate IONM is at detecting a pedicle screw cortical breach.

There have been a few case reports in the literature discussing postoperative neurologic deficit secondary to a false negative signal on IONM [11,12]. In the report by Chen et al., a T10-L5 posterior spinal fusion was performed on a 61-year-old male with thoracolumbar kyphoscoliosis. Intraoperatively, there was an initial EMG alert for a left L3 pedicle wall breach. The pedicle screw was thus removed, repositioned, and had a subsequent normal EMG signal. However, when the patient awoke from surgery, he had left lower extremity paralysis of the iliopsoas, adductor, and quadricep muscle, which had minimal recovery over time [11]. Furthermore, the report by Ohashi et al. discussed a 56-year-old female with severe kyphoscoliosis of the thoracolumbar spine who underwent a fusion from T9 to the pelvis. There was no positive signal on IONM throughout the case; however, postoperatively, the patient had weakness of her left iliopsoas and quadriceps muscles as well as hypesthesia of the anterior thigh. Postoperative neurologic deficits were attributed to the stretch of the L3 nerve root. At six months, the motor deficit resolved; however, she continued to have anterior thigh numbness after two years [12]. There are a few differences between these reports and ours. The first indication for our surgery was spondylolisthesis, compared to the cases found in the literature, which were for advanced kyphoscoliosis. This is important because during the reduction of the deformity, the nerve can become stretched and cause transient neuropraxia. Additionally, we never had an EMG signal below the threshold for a pedicle wall breach. Whereas Chen et al. did have an initial EMG signal indicating a medial wall breach.

In regards to the accuracy of IONM using EMG, a recent study from Riley et al. questions the efficacy of EMG alone and proposes the shift to a multimodal approach with EMG combined with transcranial electric motor-evoked potentials (tcMEPs) [13]. In this retrospective analysis of 479 lateral lumbar interbody fusion (LLIF) surgeries, results demonstrated a statistically significant reduction in postoperative neurologic deficits for neurophysiologist-controlled MEP (NC-MEP) monitoring vs. neurophysiologist-controlled EMG (NC-EMG) and surgeon-directed EMG (SD-EMG), both independently and combined. NC-EMP had the lowest rates of motor and sensory deficits at the immediate postoperative period, as well as at the 12-month follow-up or greater.

## Conclusions

Overall, the greatest challenge is trying to determine why these complications occur and how we can prevent them from happening in the future. Unfortunately, we still do not know whether EMG monitoring is affected by certain spinal pathology. For example, patients with severe disease could have nerve tissue that is too chronically damaged to elicit an appropriate EMG stimulus. Or perhaps, nerve injury can occur after an initial cortical breach so that any normal signal after repositioning the screw is actually invalid due to a damaged nerve. The possibility also exists that EMG alone is not the best modality in detecting these complications; therefore, a shift from the standard paradigm to a multimodal approach is best to avoid these complications. Regardless of these postulations, a false negative EMG signal could simply be due to statistical chance. In conclusion, it is important to note that IONM may not be completely accurate; therefore, it is important to be cognizant of any complications that may occur during the surgical procedure and postoperative period.
